# Quality of life in mentally ill, physically ill and healthy individuals: The validation of the Greek version of the World Health Organization Quality of Life (WHOQOL-100) questionnaire

**DOI:** 10.1186/1744-859X-8-23

**Published:** 2009-10-13

**Authors:** Maria Ginieri-Coccossis, Eugenia Triantafillou, Vlasis Tomaras, Ioannis A Liappas, George N Christodoulou, George N Papadimitriou

**Affiliations:** 1 First Department of Psychiatry, Medical School, University of Athens, Greece; 2 Hellenic Mental Health and Research Centre, Athens, Greece

## Abstract

**Objective:**

The World Health Organization Quality of Life (WHOQOL-100) questionnaire is a generic quality of life (QoL) measurement tool used in various cultural and social settings and across different patient and healthy populations. The present study examines the psychometric properties of the Greek version, with an emphasis on the ability of the instrument to capture QoL differences between mentally ill, physically ill and healthy individuals.

**Methods:**

A total of 425 Caucasian participants were tested, as to form 3 groups: (a) 124 psychiatric patients (schizophrenia n = 87, alcohol abuse/dependence n = 37), (b) 234 patients with physical illness (hypertension n = 139, cancer n = 95), and (c) 67 healthy control individuals.

**Results:**

Confirmatory factor analysis was performed indicating that a four-factor model can provide an adequate instrument structure for the participating groups (GFI 0.92). Additionally, internal consistency of the instrument was shown to be acceptable, with Cronbach's α values ranging from 0.78 to 0.90 regarding the four -domain model, and from 0.40 to 0.90 regarding the six-domain one. Evidence based on Pearson's r and Independent samples *t-*test indicated satisfactory test/retest reliability, as well as good convergent validity tested with the General Health Questionnaire (GHQ-28) and the Life Satisfaction Inventory (LSI). Furthermore, using Independent samples *t*-test and one-way ANOVA, the instrument demonstrated good discriminatory ability between healthy, mentally ill and physically ill participants, as well as within the distinct patient groups of schizophrenic, alcohol dependent, hypertensive and cancer patients. Healthy individuals reported significantly higher QoL, particularly in the *physical health *domain and in the *overall QoL/health *facet. Mentally ill participants were distinctively differentiated from physically ill in several domains, with the greatest difference and reduction observed in the *social relationships *domain and in the *overall QoL/health *facet. Within the four distinct patient groups, alcohol abuse/dependence patients were found to report the most seriously compromised QoL in most domains, while hypertensive and cancer patients did not report extensive and significant differences at the domain level. However, significant differences between patient groups were observed at the facet level. For example, regarding the *physical domain*, physically ill participants reported more compromised scores in the *pain/discomfort *facet, while mentally ill participants in the facets of *energy/fatigue, daily living activities *and *dependence on medication*.

**Conclusion:**

The findings of the study indicate that the Greek version of WHOQOL-100 provided satisfactory psychometric properties supporting its use within general and pathological populations and in the context of national and crosscultural QoL measurement.

## Introduction

During the last few decades, the measurement of quality of life (QoL) has played a key role in the evaluation of patients and treatment outcomes [1-4]. QoL measurement aims to assess the subjective nature of QoL, capturing self-perceptions of current state of life and health [5]. At present, the majority of QoL measurement tools available for assessing patients in mental or physical healthcare can be grouped into two main categories: (a) generic instruments, examining QoL as a multidimensional concept with cultural, social, psychological and health dimensions, suitable for healthy and clinical populations, and (b) disease-specific instruments, measuring specific areas of health, functioning and QoL relevant to a particular disease and treatment [6-8]. In addition, health-related QoL (HRQOL) measurements prioritise patients' point of view regarding their health, supporting thus the application of holistic, interactive and patient-centred medical practices [9].

It is worth noting that an increase of crosscultural comparisons in the field of health is directly related to QoL measurements, used as valid indicators of healthcare outcomes. Such measurements are regularly tested within specific populations, cultural settings and social environments in order to secure the validity and reliability of their use in clinical trials and research [10,11]. Consequently, in the last two decades, there has been a substantial increase in validation studies for crossculturally applicable QoL measurements, providing multiple benefits for patients, clinicians, researchers and decision makers worldwide [12,13].

### The World Health Organization Quality of Life (WHOQOL-100) questionnaire: Crosscultural QoL measurement

QoL is a broad-ranging concept affected in a complex way by the person's physical health, psychological state, personal beliefs, social relationships and the relationship to salient features of the individual's environment [14].

In the 1990s, the World Health Organization (WHO) initiated an international project aiming at the development of a comprehensive QoL measurement system for healthy and non-healthy populations, suitable for comparisons across different cultures and settings [15]. The project originally started in 15 different sites around the world, with the use of common protocols that were agreed on the basis of consensus. The diversity of national languages and the continuity of interaction among the participating countries were preconditions for collaboration, necessary for the development of a genuine crossculturally valid system of measuring QoL. Within this framework, qualitative procedures (focus groups) and quantitative and statistical methods were used for defining, refining and testing the instrument's psychometric properties [16]. The use of multilevel crosscultural methodology among the participating sites intended to safeguard conceptual and semantic equivalence between the different language versions of the instrument that could be developed. Furthermore, the specific methodology is used today as a prototype for validation protocols in developing new WHOQOL language versions.

Thus, the WHOQOL international initiative resulted in the development of a QoL measurement system, the WHOQOL-100 questionnaire, comprised of 100 items grouped into 25 facets (or factors). One of the facets measures overall quality of life/health. The remaining 24 facets were originally organised in 6 domains: (1) *physical health*, (2) *psychological health*, (3) *level of independence*, (4) *social relationships*, (5) *environment *and (6) *spirituality/religion/personal beliefs*. Each facet includes four items, rated on a five-point Likert scale, with higher scores indicating more positive evaluations of the specific facet items. Domain and facet raw scores can also be transformed onto a 0 to 100 scale, according to documented procedures included in the relevant WHO guidelines [14,16,17].

In addition, examining the possibility of grouping the WHOQOL-100 facets into a smaller number of comprehensive domains, the original six-domain structure was later reduced into a four-domain model by the WHOQOL Group, comprising: (1) *physical health *(merging the level of independence domain), (2) *psychological health *(merging the spirituality/religion/personal beliefs domain), (3) *social relationships *and (4) *environment *[13]. The facets comprising each domain are outlined later in this report (see Table 1).

**Table 1:** Discriminant validity of the World Health Organization Quality of Life (WHOQOL-100) questionnaire: Domain/facet differences between mentally ill and physically ill participants (Independent samples *t*- test)

**WHOQOL-100 domains/facets**	**Mentally ill (n = 124)**	**Physically ill (n = 234)**	***t*-test**	***p *value**
Physical health	59.06 (16.76)	61.44 (17.84)	1.22	0.221
Pain and discomfort	62.61 (24.80)	55.80 (24.13)	-2.51	0.012
Energy and fatigue	52.06 (20.91)	57.79 (20.10)	2.52	0.012
Sleep and rest	64.14 (27.17)	62.60 (27.19)	-0.510	0.610
Mobility	67.99 (24.39)	67.40 (22.95)	-.226	0.821
Activities of daily living	55.91 (22.81)	65.37 (20.12)	4.03	0.000
Dependence on medication	52.85 (26.88)	61.58 (27.95)	2.84	0.005
Working capacity	57.30 (25.93)	61.86 (24.21)	1.65	0.100
Psychological health	56.66 (18.97)	64.74 (13.21)	4.70	0.000
Positive feelings	45.66 (20.99)	51.89 (18.14)	2.92	0.004
Thinking, earning, memory and concentration	58.18 (21.12)	67.84 (15.80)	4.86	0.000
Self-esteem	58.65 (23.05)	68.46 (16.81)	4.59	0.000
Bodily image and appearance	65.74 (23.99)	70.76 (21.11)	2.03	0.042
Negative feelings	46.85 (20.85)	49.66 (22.93)	1.13	0.258
Spirituality/religion/personal beliefs	58.31 (23.63)	67.73 (16.63)	4.38	0.000
Social relationships	54.05 (17.36)	65.32 (16.85)	5.95	0.000
Personal relationships	59.61 (20.59)	75.22 (17.42)	0.756	0.000
Social support	56.50 (22.81)	64.95 (22.37)	3.37	0.001
Sexual activity	45.93 (23.44)	53.14 (22.42)	2.74	0.006
Environment	59.75 (12.28)	58.76 (13.18)	-0.691	0.490
Physical safety and security	60.70 (18.56)	51.81 (20.08)	-4.07	0.000
Home environment	64.73 (18.19)	66.64 (17.85)	0.951	0.342
Financial resources	48.88 (25.07)	59.24 (26.32)	3.59	0.000
Health and social care: availability and quality	62.85 (17.24)	55.98 (18.40)	-3.42	0.001
Opportunities for acquiring new information and skills	56.77 (17.67)	56.01 (15.39)	-0.418	0.676
Participation in and opportunities for recreation/leisure	54.88 (19.85)	53.73 (18.93)	-0.538	0.591
Physical environment	64.51 (18.69)	63.11 (18.99)	-0.668	0.505
Transport	64.11 (22.90)	63.51 (23.75)	-0.229	0.819
Overall quality of life and general health	50.00 (22.47)	57.61 (18.26)	3.45	0.001

Values are mean (SD) unless otherwise stated. *p *< 0.05.

SD = standard deviation.

The six-domain WHOQOL-100 model has been used in several validation studies, wherein satisfactory psychometric properties were produced, as in the case of the first Dutch validation study (Cronbach's α 0.71 to 0.93 across the six domains) [18]. Additionally, its application in the UK revealed significant QoL outcomes for people attending a pain management programme, indicating satisfactory overall internal consistency and reliability for most facets and domains except for the pain and discomfort facet, which had a marginal outcome [19].

Furthermore, the WHOQOL-100 four-factor model has been proposed in a number of studies as a more suitable fit than the original six-domain structure. For example, examining the equivalence between the Hindi and English versions of the WHOQOL-100 in north India, the results of confirmatory factor analysis suggested a satisfactory fit for a four-factor structure (Comparative Fit Index (CFI) = 0.82) in and across both language versions [20]. Similarly, using the WHOQOL-100 in patients with chronic diseases and in their caregivers in China, the results of principal component analysis produced four factors accounting for 61% of the total variance [21]. Additionally, according to a recent Dutch validation study with a population of adult psychiatric outpatients, a four-factor structure was revealed with satisfactory CFI (0.90), only with the exception of two facets (physical environment and transport), which were omitted from the instrument [22].

Since the development of the WHOQOL-100, great emphasis has been given to the validation of WHOQOL in different language versions, with the view to enhance the possibility of performing valid crosscultural comparisons. The WHOQOL-100 has been described as a valid and reliable instrument for use among ill and healthy population groups [10,20]. Its wide application across countries and populations may be observed in several studies, for example: (a) diabetic patients in Croatia, whereby the obtained Cronbach's α values for the domains were found satisfactory (physical 0.95, psychological 0.89, social 0.76 and environmental 0.92), indicating that the instrument was reliable and valid for this particular population [23]; (b) psychiatric patients in Turkey, where good internal consistency was also obtained (α range: 0.67 to 0.87 across domains) [24]; (c) depressed patients in the UK and Argentina, demonstrating the functionality of the WHOQOL-100 to identify reduced QoL in this population [25]; (d) individuals in India, where a Hindi version of WHOQOL-100 was considered an appropriate instrument for comprehensively assessing QoL in healthcare settings [26]; (e) psychiatric patients in Italy, where the usefulness of WHOQOL-100 was observed in assessing QoL in schizophrenic patients and comparing their reports with their proxies, using the QOL-P (derived from WHOQOL-100) [27]; and (f) traumatised Iranian refugees resettled in Sweden, where the instrument was found valuable in assessing the relationship between QoL, psychopathological manifestations and coping [28].

Regarding the instrument's responsiveness to treatment change, QoL changes were identified in chronic pain patients in the UK who participated in a pain management programme [19], in moderately depressed patients following medical treatment [29], in a group of alcoholic patients in Greece following a specialised in-hospital detoxification programme [30], as well as in a group of American women after childbirth [31].

### Aim of the study and research hypotheses

The aim of the present study was to examine the validity and reliability of the WHOQOL-100 Greek version and assess its suitability for identifying differences in QoL between mentally ill, physically ill and healthy individuals.

In the context of examining discriminant validity, the authors made the assumption that distinct differences would be found between healthy participants and patient groups. Specifically, in several validation studies poorer QoL has been reported in physically ill populations, including patients with chronic fatigue syndrome and patients with different types of physical illness [18,5].

Furthermore, QoL differences were assumed between psychiatrically ill and physically ill participants due to the fact that, in the body of relevant literature, mentally ill individuals across age groups are found to report a substantially compromised QoL in different domains. In the present study, it was assumed that lower QoL scores would be observed in the WHOQOL-100 *social relationships *and *psychological health *domains [32,33].

It is further noted that investigation of QoL differences between patients with psychiatric disorders and those suffering from organic or physical illness is limited and not systematically reported in the international literature. Thus, for instance, findings from a validation study in China have shown that schizophrenic patients differ in QoL from various groups of physically ill patients [21]. Additionally, in the context of Dutch, Turkish and Argentinean WHOQOL-100 validation studies, mentally ill individuals, including schizophrenic, depressed or patients with other psychiatric disorders, have reported several QoL impairments [22,24,25].

In addition, regarding mentally ill participants, QoL differences were assumed to exist between two distinct diagnostic categories: schizophrenic and alcohol abuse/dependent patients. Specifically, it was expected that the latter group of patients would report poorer QoL in several or most of the WHOQOL-100 domains because of recent consumption-related psychopathology and multiple acquired deficits in physical and psychological health, in social life, family, work and financial well-being [34-37].

Regarding physically ill individuals, the assumption was made that participants with hypertension and cancer would report reduced QoL in physical and mental health related domains. Regarding WHOQOL domains and facets, it was hypothesised that QoL deficits would probably be obtained in the facets of *pain/discomfort *(in the *physical health *domain) and in experiencing *positive feelings *(in the *psychological health *domain). Recent studies indicate that both of these clinical populations were found to report reduced physical and emotional well-being: hypertension symptoms seem to have a greater negative impact on physical related and mental related scores, while patients with different types of cancer have reported compromised emotional well-being (with the use of different QoL instruments) [38,39].

With reference to the examination of convergent validity, using other relevant validated instruments, it was assumed that specific WHOQOL-100 domain scores would relate to scores obtained from similar scales, such as the Life Satisfaction Inventory (LSI), or similar subscales, such as those included in the General Health Questionnaire (GHQ-28). In this respect, it was expected that the WHOQOL-100 *overall QoL/health *facet would correlate with the GHQ-28 and LSI total scores. Additionally, the *physical health *domain was expected to show high correlations with the GHQ-28 somatic symptoms and the anxiety/insomnia subscales; the *psychological health *domain was hypothesised to demonstrate high correlations with the GHQ-28 severe depression subscale, while the *social relationships *domain would correlate with the total LSI score.

Concerning the *environment *domain, comprising a variety of facets referring to different aspects of an individual's environment, it was hypothesised that rather low correlations would be produced with the GHQ-28 subscales or low to moderate correlations with the total LSI score. This is proposed on the basis that these two instruments do not include similar items examining perceived environmental aspects. At best, the *environment *domain would show a moderate correlation with the total LSI scale score, which contains two items (hobbies and financial status) that seem to have an affinity with two facet items of the *environment *WHOQOL-100 domain that is *participation in recreation/leisure *and *financial resources *(see section on Instruments and specifically the description of the LSI questionnaire).

Finally, it was assumed that within a 3 to 4-week reassessment period, the domain values produced by the healthy participants would demonstrate satisfactory correlations of test/retest reliability, similarly to other validation studies, such as the Canadian and the US versions of WHOQOL-100 [31,40].

## Methods

### Participants

The sample was recruited following the guidelines of the WHO protocol for New Centers, according to which it was recommended to include a minimum of 250 individuals with a disease or impairment and 50 'well persons' [41]. Recruitment of participants was conducted on the basis that chronically ill individuals, either with physical or psychiatric illness, would be suitable for a validation study investigating discriminatory QoL differences and deficits. Thus, a total sample of 425 Caucasian Greek individuals, who voluntarily participated in the study, comprised 3 groups: (a) participants with psychiatric disorders (n = 124), (b) participants with physical illness (n = 234), and (c) healthy participants as a control group (n = 67). Comparisons between patients with physical and mental disorders and with a healthy control group have been reported in the context of the Danish WHOQOL validation study [42].

Regarding mentally ill participants, two distinct groups of patients were included: (1) chronic psychiatric outpatients diagnosed within the schizophrenia-psychotic spectrum (n = 87), who were using community mental health services and receiving antipsychotic medication (inclusion criteria for these patients identified the absence of major physical or neurological disorders), and (2) psychiatric inpatients, who were consecutively admitted with a diagnosis of alcohol abuse/dependence (n = 37), and were hospitalised within a 5-week detoxification programme [30]. Both groups were recruited from the Athens University Psychiatric Hospital and were all confirmed as having fulfilled the relevant criteria for their particular disorder according to the Diagnostic and Statistical Manual of Mental Disorders, fourth edition (DSM-IV) [43].

With reference to the physically ill participants, two different groups were included: (1) hypertensive patients diagnosed by their physicians with moderate or severe hypertension (n = 139), and (2) cancer patients, including approximately 50% women with breast cancer, and none of them in palliative care or chemotherapy within the previous year (n = 95). Inclusion criteria for both groups of physically ill participants identified patients who were undergoing treatment during the previous 5 years. Recruitment of patients took place in relevant outpatient units at public general hospitals located in the same area as the above-mentioned psychiatric services.

Finally, a group of healthy participants was recruited (n = 67), identified as a gold standard group, unmatched for sociodemographic variables. Specifically, healthy participants were younger and more educated than the participants of the illness groups (Table 2). They were recruited from the administrative personnel of public health and research services of the same area. Recruiting healthy individuals as a control group provided the opportunity to compare QoL variables between healthy and clinical groups, and test the discriminatory power of the instrument within these populations. Furthermore, the healthy control group was used for test/retest reliability, requiring a re-administration of the instrument within 3 to 4 weeks on the basis that significant changes were not expected to occur in the elapsed time.

**Table 2:** Sociodemographic characteristics for physically ill, mentally ill and healthy participants

	**Physically ill (n = 234)**	**Mentally ill (n = 124)**	**Healthy (n = 67)**
Age	60.71 (11.11)	40.79 (11.88)	32.75 (8.12)
Gender	75 (32.1)	83 (66.9)	20 (29.9)
*Male/female*	159 (67.9)	41 (33.1)	47 (70.1)
Years of education	9.15 (3.83)	11.25 (3.55)	14.97 (2.65)
Marital status:			
*Single*	17 (7.3)	72 (58.1)	30 (44.8)
*Married/cohabitating*	168 (71.8)	35 (28.2)	34 (50.7)
*Postmarital (separated, divorced, widowed)*	49 (20.9)	17 (13.7)	3 (4.5)

Values are mean (SD) or n (%).

SD = standard deviation.

In accordance with the study's protocol, all subjects were volunteers. They had been informed of their rights to refuse or discontinue participation and each individual signed a consent form, according to the ethical standards of the Helsinki Declaration of 1975, as revised in 1983. Ethical approval for the study was obtained from the scientific committee of the Department of Psychiatry of the University of Athens. All participants were screened for their ability to take part in the study, including literacy.

### Instruments

The total sample of participants completed the selected self-report questionnaires, including WHOQOL-100, LSI and GHQ-28, which were administered by appropriately trained healthcare personnel and under standardised conditions. Health and life satisfaction measurements were selected on the basis of being suitable for performing validity testing for QoL.

### The WHOQOL-100 Greek pilot version

The instrument was translated following a multifaceted procedure in accordance with the guidelines documented by WHO [44]. In addition, facet structure, comprehensiveness, linguistic and cultural suitability were examined with the use of focus group methodology [45]. The instrument's sensitivity to clinical change has been already investigated in a pre/post design for patients following an alcohol detoxification programme, yielding highly satisfactory outcomes [30]. Higher facet or domain scores are indicative of more positive perceived QoL evaluations.

### LSI

This is a generic 13-item measurement tool, previously validated in Greek populations and revealing a 4-factor model (general well-being, family life, financial status/occupation, and mental and general health) [46,47]. The instrument has demonstrated good internal consistency (Cronbach's α 0.82), including items that examine the level of satisfaction regarding different aspects of an individual's life: physical state, mental state, psychological health, occupation, financial status, relationships with partners, sexual life, family life, role in the family, friends and acquaintances, hobbies, physical appearance, and general QoL. A higher total score is indicative of greater self-reported life satisfaction.

### GHQ-28

This is a widely used self-report questionnaire of general health, designed by Goldberg for the purpose of detecting mental health problems in non-clinical settings [48]. The instrument can identify short-term changes in mental health and is often used as a screening tool for psychiatric cases in a number of medical settings including general practice. The GHQ 28-item version, which was used in this study, has been validated demonstrating good psychometric properties within Greek populations (internal consistency, validity with indices of sensitivity, specificity, positive predictive value, negative predictive value and overall misclassification rate) [49]. The GHQ scale provides a total score, as well as separate scores for four subscales regarding health: (a) somatic symptoms, (b) anxiety and insomnia, (c) social dysfunction and (d) severe depression. A lower score is indicative of a more positive self-perception regarding health. In the context of the present study, GHQ-28 scores have been reversed in order to correspond with the direction of all the scores in the above-mentioned questionnaires.

### Statistical analyses

Data sets were analysed using SPSS for Windows, V.13.0 (SPSS, Chicago, IL, USA). A range of statistical tests were used, including confirmatory factor analysis. Internal consistency was examined by calculating the Cronbach's α for each domain, both in the six-domain and four-domain models and across the three participating groups (healthy, mentally ill, and physically ill). Independent sample *t*-tests were used, in order to identify the instrument's ability to discriminate between healthy/non-healthy and between mentally ill/physically ill participants. Additionally, analysis of variance (ANOVA) (with *post hoc *Scheffe) was used to test for differences among the distinct patient groups (schizophrenic, alcoholic, hypertension, cancer). The Pearson's *r *was used to test the instrument's ability to converge and harmonise with other instruments measuring similar constructs. Thus, convergence was examined between the WHOQOL-100, the subscales of the GHQ-28 and the total scores of GHQ-28 and LSI scales in the total sample. Finally, to determine the test/retest reliability of the instrument, Independent samples *t*-tests were used to confirm that no significant differences were evident between the initial and the subsequent assessment (3 to 4 weeks) in the healthy group participants. Pearson's *r *was also used to identify consistency of responses between the two measurements.

## Results

Using the Kolmogorov-Smirnov test of goodness of fit, the variable scores in the total sample appeared to have non-normal distributions. However, when data was examined separately in each participating group, it was generally found to conform to a normal distribution.

### Subjects

Regarding sampling, the degree of control on sociodemographic variables, which is required in clinical trials, is not necessary for validation testing. It is generally sufficient to provide evidence that QoL scores reflect adequately that ill participants tend to report lower QoL scores than healthy individuals. This is mentioned in the WHO protocol regarding psychometric testing for new WHOQOL versions [41]. Thus, sociodemographic differences were expected to be observed among the participating groups in the present study. Characteristics of the three groups are displayed in Table 2.

### Structure of WHOQOL-100

Confirmatory factor analysis was performed demonstrating that the four-domain model of physical health, psychological health, social relationships and environment was a good fit for the specific populations studied, accounting for 60% of the total variance. GFI indices demonstrated index values of 0.92, therefore meeting the required criteria (values of 0.90 or higher are considered a reasonable level of fit for the model). Additionally, model χ^2 ^testing revealed no significant differences between the hypothesised structure and the observed data (*p *> 0.05).

### Internal consistency

Internal consistency of the instrument was examined using Cronbach's α coefficient [50]. It was applied to both six- and four-domain models and the *overall QoL/health *facet, across the three participating groups (healthy, mentally ill, and physically ill). In the four-domain model, satisfactory scores were obtained for each subsample, ranging from 0.78 to 0.90, indicating good internal consistency for all domains and the *overall QoL/health *facet (Table 3). Internal consistency was also examined in the six-domain model producing domain values ranging from 0.40 to 0.90 (Table 4). Comparing the α values between the two models, lower values were identified in the six-domain model regarding the physical health domain (the value for the healthy group was 0.40, the physically ill 0.50, and for the mentally ill 0.65).

**Table 3:** Cronbach's α coefficients for the four-domain World Health Organization Quality of Life (WHOQOL-100) questionnaire in physically ill, mentally ill and healthy participants

**WHOQOL four domains**	**Physically ill (n = 234)**	**Mentally ill (n = 124)**	**Healthy (n = 67)**
Physical health	0.86	0.80	0.86
Psychological health	0.78	0.87	0.79
Social relationships	0.85	0.84	0.85
Environment	0.90	0.90	0.90
Overall QoL/health	0.82	0.83	0.83

QoL = quality of life.

**Table 4:** Cronbach's α coefficients for the six-domain World Health Organization Quality of Life (WHOQOL-100) questionnaire in physically ill, mentally ill and healthy participants

**WHOQOL six domains**	**Physically ill (n = 124)**	**Mentally ill (n = 234)**	**Healthy (n = 67)**
Physical health	0.50	0.65	0.40
Psychological health	0.70	0.80	0.60
Level of independence	0.73	0.85	0.80
Social relationships	0.85	0.84	0.85
Environment	0.90	0.90	0.90
Spirituality/religion/personal beliefs	0.80	0.90	0.90
Overall QoL/health	0.82	0.83	0.83

QoL = quality of life.

### Discriminant validity

Differences regarding the WHOQOL-100 domain scores were investigated between: (a) healthy participants and the total population of ill participants, (b) between participants with psychiatric disorders and those with physical illness, and (c) across four distinct clinical groups (schizophrenic, alcoholic, hypertension, and cancer). Independent samples *t*-tests and one-way ANOVA (with *post hoc *Scheffe) demonstrated the instrument's ability to discriminate between the participating groups (healthy, mentally ill and physically ill), and within the four patient groups. Additionally, discriminant validity was examined for gender and age.

It was observed that the healthy control group achieved significantly higher mean scores than the total patient population (mentally ill and physically ill), for all domains except the *environment *(Table 5). Differences in scores are particularly evident for the *physical health *domain, and the *overall QoL/health *facet, demonstrating that healthy participants reported significantly higher scores in these two health-related QoL domains, which may be considered as good indicators of health.

**Table 5:** Discriminant validity of the World Health Organization Quality of Life (WHOQOL-100) questionnaire: Domain differences between healthy and total patient group participants (Independent samples *t*- test)

**WHOQOL domains**	**Healthy (n = 67)**	**Total patient group (mentally/physically ill; n = 358)**	***t*-test**	***p *value**
Physical health	76.27 (13.07)	60.62 (17.49)	-4.44	0.00
Psychological health	69.99 (12.00)	61.93 (15.90)	-3.58	0.00
Social relationships	72.57 (14.00)	61.42 (17.83)	-4.84	0.00
Environment	57.07 (11.39)	59.10 (12.87)	1.20	NS
Overall QoL/health	69.12 (15.14)	54.97 (21.12)	-5.47	0.00

Values are mean (SD) unless otherwise stated. *p *< 0.05.

NS = not significant; QoL = quality of life; SD = standard deviation.

In addition, significant differences regarding the WHOQOL domain and facet mean scores were identified between mentally ill and physically ill participants in a number of facets and across all, with the exception of the *physical health *and *environment *domains (Table 1). Regarding facet scores within the *physical health *domain, it is observed that physically ill participants reported statistically compromised scores in the *pain/discomfort *facet, as expected, while mentally ill participants reported compromised scores in the facets of *energy/fatigue, daily living activities *and *dependence on medication*.

Regarding the *psychological health *domain, mentally ill participants indicated significantly more compromised scores in all but the *negative feelings *facet, while, as expected, both psychiatrically and physically ill participants reported considerable distress as seen in the considerably low scores in the *negative feelings *facet.

For the domain of *social relationships*, mentally ill participants indicated significantly lower scores than physically ill in all facets, supporting the proposed hypothesis that psychiatric participants would report QoL deficits, particularly regarding their social well-being.

Finally, in reference to the *environment *domain, physically ill participants indicated lower scores in the *safety/security *and *health services *facets, while psychiatrically ill participants reported lower scores in the *financial resources *facet, as expected. The remaining facets did not provide significant differences between these two clinical groups. Regarding the *overall QoL/health *facet, mentally ill participants reported significantly lower scores than the physically ill, as expected.

Further, one-way ANOVA and *post hoc *Scheffe were used to examine discriminant validity within the four distinct patient groups, wherein a number of QoL differences were identified (Table 6). It was observed that WHOQOL-100 domain mean differences between the two physically ill groups (cancer and hypertensive) were not as great as they appeared to be between the psychiatric groups (schizophrenic and alcoholic). Additionally, the lowest domain mean scores were observed in the alcohol abuse/dependence group, particularly in the *overall QoL/health *facet. The calculation of F values provided evidence of systematic differences across groups particularly in the *overall QoL/health *facet. The Scheffe test was used for multiple comparisons between the four groups. In the case of cancer and hypertensive participants, the results showed that QoL domain differences between these two patient groups are not statistically significant. By contrast, significant differences were observed between schizophrenic and alcoholic participants, with the latter presenting lower QoL scores (*p *< 0.001).

**Table 6:** Differences in World Health Organization Quality of Life (WHOQOL-100) questionnaire domain scores among four patient groups by analysis of variance (ANOVA)

**WHOQOL-100 domains**	**Schizophrenia (n = 87)**	**Alcohol (n = 37)**	**Hypertension (n = 139)**	**Cancer (n = 95)**	**F**	***p *value**
Physical health	61.45 (14.76)	53.43 (19.81)	60.44 (17.57)	62.90 (18.23)	2.73	0.044
Psychological health	59.08 (18.66)	50.95 (18.71)	64.37 (12.82)	65.27 (13.81)	9.98	0.000
Social relationships	55.44 (17.74)	50.78 (16.19)	63.64 (16.63)	67.78 (16.96)	13.70	0.000
Environment	59.02 (12.26)	61.45 (12.34)	56.23 (13.33)	62.46 (12.10)	5.04	0.002
Overall QoL/health	56.34 (20.71)	35.07 (19.33)	57.68 (17.34)	57.55 (19.62)	20.33	0.000

Values are mean (SD) unless otherwise stated. *p *< 0.05.

QoL = quality of life; SD = standard deviation.

Given the diverse age ranges across the different groups of participants (range: 18 to 82), the instrument's ability to highlight age differences was investigated. Thus, participants who were younger than 45 years old were compared to those above 45. The cut-off point for age was set in accordance with the WHO protocol concerning the validation of new language versions [41]. Participants under 45 indicated higher scores in the *environment *domain (Mann-Whitney test *p *< 0.05, z value 1,97). Additionally, a non-significant tendency was observed in the *physical health *domain.

Investigating gender differences in the total population of participants across WHOQOL-100 domain scores, no significant differences were found between male and female participants.

### Convergent validity

Convergent validity was investigated using the Pearson's *r*, with results supporting the proposed assumptions (Table 7). Using the whole sample (healthy, mentally ill, and physically ill), the instrument's *physical health *domain was highly related to the GHQ-28 subscales of somatic symptoms, anxiety/insomnia, and social dysfunction, as well as to the GHQ-28 total score. Additionally, high correlations were observed between the WHOQOL-100 *psychological health *domain and the following: (a) the GHQ-28 severe depression subscale, (b) the GHQ-28 total score, and (c) the total LSI score. Moreover, in agreement with the proposed hypotheses, a moderate relationship was obtained between the WHOQOL-100 *social relationships *domain and the GHQ-28 social dysfunction subscale, reflecting a moderate content affinity between them. Further, the WHOQOL-100 *social relationships *domain yielded a significantly high correlation with the total LSI score.

**Table 7:** Convergent validity: Correlations between World Health Organization Quality of Life (WHOQOL-100) questionnaire domains, General Health Questionnaire (GHQ-28) subscales and total scores of GHQ-28 and Life Satisfaction Inventory (LSI) (Pearson's correlation coefficient) for the total sample (n = 425)

**WHOQOL-100 domains**	**GHQ-28 somatic symptoms**	**GHQ-28 anxiety/insomnia**	**GHQ-28 social dysfunction**	**GHQ-28 severe depression**	**GHQ-28 total score**	**LSI total score**
Physical health	0.63^a^	0.57^a^	0.57^a^	0.52^a^	0.60^a^	0.41^a^
Psychological health	0.47^a^	0.47^a^	0.49^a^	0.66^a^	0.64^a^	0.48^a^
Social relationships	0.33^a^	0.38^a^	0.37^a^	0.45^a^	0.45^a^	0.74^a^
Environment	0.09	0.26^a^	0.17^a^	0.22^a^	0.22^a^	0.47^a^

Overall QoL/health	61^a^	57^a^	0.53^a^	0.60^a^	0.67^a^	0.78^a^

^a ^*p *< 0.01.

QoL = quality of life.^a^

Finally, the WHOQOL-100 *overall QoL/health *facet yielded the highest correlations with the total GHQ-28 and LSI scores. The WHOQOL-100 *environment *domain demonstrated low correlations with all GHQ-28 health subscales and as hypothesised, a moderate correlation with the total LSI score (r = 0.47).

### Test/retest reliability

The healthy group was reassessed for test/retest reliability analysis. An Independent samples *t*-test indicated no statistical differences in domain mean scores between the two administrations of the WHOQOL-100 instrument. Test/retest reliability was also confirmed by the use of the Pearson correlation, which demonstrated consistency of responses between first and second administration (r = 0.66, *p *< 0.01).

## Discussion

The results of the present study provide evidence on the psychometric properties of the WHOQOL-100 Greek version in terms of structure, internal consistency, discriminant and convergent validity, and test/retest reliability. The overall findings were observed to support the proposed hypotheses.

Exploring the factor structure of the WHOQOL-100 in the Greek version, a four-factor solution was identified as a satisfactory fit. This finding is in agreement with international results showing that the WHOQOL-100 four-factor model may be a reasonable fit across different cultures [10,12,13]. Both the six- and the four-domain models have been used reliably in international QoL research. The four-domain model was employed in several validation studies with general and clinical populations [20-22].

With regards to the instrument's internal consistency, it was generally well supported, with satisfactory *alpha *scores in the four domains across the three groups, as shown in Table 3, indicating that the instrument is an internally reliable tool for the assessment of quality of life in Greek populations. In the six-domain structure, *alpha *scores were satisfactory in all but the *physical health *domain (Table 4). It is noted that in the four-domain model, the domain of *physical health *contains more items, which were obtained due to the merging of the items of the level of independence domain within the *physical health *domain. Added items may account for more satisfactory *alpha *scores observed in the composite *physical health *domain.

Investigating the instrument's ability to discriminate between healthy and non-healthy populations, the findings are in accordance to the hypotheses demonstrating that healthy participants reported considerably higher scores in several domains, specifically in the *physical health *domain and the *overall QoL/health *facet (Table 5). This was expected, since the healthy control group was considered as a positive standard on the basis that participants were healthy, younger and more educated than the participants in the two clinical groups. It can be argued that in this case, the domain of *physical health *and the facet of *overall QoL/health *may stand as discriminatory indicators between healthy and non-healthy populations. The above findings are in agreement with several WHOQOL-100 validation studies, which indicate significantly higher QoL values for healthy cohorts in the *physical health*, as well as the *psychological health *domains [5,20,24,51].

In addition, assumptions regarding differences between physically ill and mentally ill participants were confirmed, with the latter experiencing significantly lower QoL in several domains (Table 1). As expected, psychiatric patients reported considerable interpersonal and social deficits, as well as lack of social support as measured by the facets of WHOQOL-100 *social relationships *domain. It is argued that this domain proves to be of high discriminatory value for ill mental health, reflecting in particular the deficits of patients who suffer from chronic and debilitating mental disorders. This finding is in agreement with other WHOQOL outcomes indicating that psychiatric patients, such as the schizophrenic, experience poor social well-being and lack of social network support [52].

According to the findings, participants with mental disorders reported more extended deficits in most of the facets of the *psychological health *domain, as well as poorer *overall QoL/health*. This is in agreement with previous WHOQOL-100 studies, wherein there was evidence of poor psychological well-being in depressed patients [53]. In the present study, mentally ill participants indicated deficits in their emotional and cognitive functioning and, as expected, they reported poorer scores in the respective facets of *self-esteem, difficulties in thinking, learning, memory and concentration*, as well as in their capacity for endorsing *spiritual beliefs *(Table 1).

It is noteworthy that both psychiatric and physically ill groups reported a high level of *negative feelings *in the respective facet. As originally thought, cancer and hypertensive patients may have poor emotional well-being, which corresponds to their reports of experiencing high levels of *negative feelings*, such as depression, anxiety, anger or distress (as examined in the respective WHOQOL facet). It seems that physically ill patients indicated experiencing dysfunctional feelings induced by their condition of health. However, these feelings did not affect their overall psychological functioning. By contrast, psychiatric patients did experience several psychological deficits, such as lower levels of self-esteem and cognitive difficulties.

Investigating further differences in perceived physical health, significant differences between physically ill and mentally ill participants were obtained particularly at the WHOQOL facet level. Thus, while differences were not observed regarding the domain level of *physical health*, significant differences were identified within-domain facets. Specifically, psychiatrically ill participants, as it was expected, reported experiencing a lower *level of energy*, more difficulty in carrying out *daily living activities*, and a higher level of *dependence on medication *(Table 1). Moreover, it is noted that the facet of *pain and discomfort *significantly differentiated the two patient populations (physically ill versus mentally ill). As expected, cancer and hypertensive participants experienced a higher level of physical pain affecting their everyday life. It should be thus pointed out that while total scores in a specific domain may not provide sufficient group differences, facet scores within domains may, by contrast, reveal important health-related QoL deficits, which may provide distinctions between different diagnostic patient groups.

Regarding physical well-being, it is argued that both groups of mentally ill and physically ill participants may experience physical symptoms that can compromise their QoL. For example, psychiatric patients frequently report complaints of persistent and frustrating nature, such as sleep difficulties or somatic pain, and identify several physical manifestations comorbid to psychiatric disorders [54]. It is thus possible that the psychiatric participants experienced poor physical health that may correspond to the physically ill participants' negative health perceptions, due to the severity of their illness (cancer, severe hypertension). On this occasion, it is recommended that psychiatric healthcare may develop specialised interventions to address physical needs and provide relevant promotion programs, in order to enhance physical health and well-being in mentally ill individuals.

To highlight this point, neglected healthcare needs of psychiatric patients have been previously reported in a study using focus group interviews. Accordingly, schizophrenic participants identified physical well-being as a priority issue of their QoL, indicating that their physical health was worse than the health condition of terminally ill patients who are at the end stage of their illness [55]. Further analysis of differences between physically ill and mentally ill participants is beyond the scope of the present study and it could be obtained with the analysis of the GHQ-28 data.

Regarding the *environment *domain, significant differences were identified between the two patient populations in reference to related facets. Specifically, as expected, mentally ill individuals indicated worse *financial conditions *than physically ill participants (Table 1). It is well known that a great number of psychiatric patients are not able to maintain a stable and productive work status. Also, psychiatric participants in this study indicated enjoying a greater availability and better quality of health services and social care, as well as experiencing more safety and security regarding their environment. These results may be group-specific reflecting the effect of mental healthcare and psychosocial support that participants were provided with at the time of the study. As mentioned in the methodology section, the psychiatric patients were either attending an outpatient rehabilitation programme or were hospitalised in a specialised inpatient detoxification unit. In both cases, patients were provided with psychosocial services that may create feelings of safety and induce favourable perceptions of environmental factors, such as access to and quality of health services.

Overall, the findings reveal the presence of QoL differences between the two participating clinical populations, each indicating, respectively, illness-specific QoL deficits including compromised emotional and social well-being, poorer physical well-being, and particular environmental restrictions. These areas of reduced QoL need to be addressed separately for mentally ill and physically ill individuals in the context of healthcare services. Moreover, it is argued that the development of a comprehensive quality of life agenda may be useful in order to provide disease-specific, patient-focused and individualised QoL rehabilitation services for individuals suffering from chronic illness, either mental or physical [32,39,51,56,57].

On investigating further the discriminatory ability of WHOQOL-100 among the four distinct patient groups, as expected, marked QoL deficits were reported by the alcohol abuse/dependence group of patients on most WHOQOL domains and the *overall QoL/health *facet. Again, the findings could be useful in both clinical practice and research. For example, QoL deficits effected in relation to the disorder or condition under investigation, in this case alcohol dependence, can be identified as indicators for planning interventions, while QoL gains can be assessed following abstinence, treatment or psychosocial programmes [30,36].

Concerning cancer and hypertensive patients, very slight QoL domain differences were identified between these groups, as seen in Table 6 (the Scheffe test did not provide significant differences). It could be argued that this finding may reflect that both patient groups could be sharing common health perceptions or beliefs concerning suffering from a serious life-threatening illness (cancer or hypertension), which at the same time is considered to be a chronic one. It is noted that such perceptions may characterise cancer patients as well as hypertensive participants especially those diagnosed with severe hypertension. In this case, patients receive medication and are aware of being at high risk for serious cardiovascular problems.

Investigating QoL differences between age groups, younger individuals reported better QoL regarding their *physical health *and *environment*. It could be argued that certain facets in the *environment *domain, such as *ability for recreation *and *ability for acquiring new information and skills*, could distinguish younger from older participants. Associations between age and QoL have been reported in the relevant literature, for example higher age coinciding with less satisfaction with one's social relationships [22].

Regarding gender, no significant differences were identified in the total sample. Taking into consideration that gender differences in QoL are not systematically evident, it would be beneficial to explore such differences in future studies across different participating groups. Gender differences may be found at the level of specific facets or items (for example items on negative emotions, or anxiety and depression) on the basis that there is evidence from previous studies, which show that women in general tend to report higher levels of depression and anxiety [58,59].

In reference to the WHOQOL-100 convergent validity, the findings provided evidence of satisfactory correlations between QoL, life satisfaction and self-reported health, supporting our hypotheses that specific QoL domains would show association to related subscales in other instruments. Thus, convergent validity, as tested in the total sample, indicated that the WHOQOL-100 *overall QoL/health *facet significantly correlated with overall assessments of the LSI and GHQ-28 instruments. In addition, as expected, strong correlations were found between: (a) the *physical health *domain (examining physical symptoms and well-being), and the related GHQ-28 subscale of somatic symptoms, as well as the overall assessment of GHQ-28; (b) the *psychological health *domain (examining psychological well-being) and the related GHQ-28 severe depression subscale, as well as the overall GHQ-28 measurement; and (c) the *social relationships *domain (examining factors of social support, personal and social relationships) and the overall LSI comprising items with similar content (Table 7).

Finally, the observed low correlations between the *environment *domain and the GHQ-28 and LSI measurements were expected, due to little conceptual affinity with these measurements. The *environment *domain is comprised of several factors examining a number of different environmental aspects. However, in the GHQ-28 and LSI measurements, a component of equivalent conceptual broadness is not included (which would be necessary for a robust convergence testing).

Regarding test/retest reliability, the findings supported the ability of the WHOQOL-100 to provide similar responses when readministered within a 3 to 4-week period to a healthy group of individuals who had not undergone any clinical intervention, or were not expected to have any changes in their lifestyle or health during this interval.

Overall, the findings of the present study converge with the results provided by previous WHOQOL-100 validation studies on different language versions. For example, the WHOQOL-100 US version, which was tested in a size sample of 443 adults (including 251 chronically ill participants, 128 healthy adults, and 64 childbearing women), demonstrated a satisfactory level of internal consistency (*alpha *range: 0.82 to 0.95 across the 6 domains), as well as reproducibility (intraclass correlation coefficient (ICC) range: 0.83 to 0.96 at 2-week retest interval), responsiveness to change in clinical conditions (as shown by predicted score change (effect size) in women after childbirth), convergent validity (with the use of 2 questionnaires, the Short Form-36 questionnaire, and the Subjective Quality of Life Profile), and discriminant validity between the diverse groups of that study [31].

Similarly, regarding the validation study in the UK using a sample of 106 chronic pain patients, the WHOQOL-100 demonstrated good overall internal consistency reliability for all facets (except for the pain and discomfort facet, which was marginal), good concurrent validity, as well as very good responsiveness to clinical change [19]. Furthermore, the WHOQOL-100 Dutch version, which was tested in a sample of 220 individuals (147 healthy people and 73 chronic fatigue syndrome patients), demonstrated a fairly good internal consistency (*alpha *range: 0.71 to 0.93 across the 6 domains), a good construct validity using a number of instruments including the Sickness Impact Profile and the Fatigue Impact Scale, as well as discriminatory capacity between the healthy and the chronic fatigue syndrome patients [18].

Additionally, the WHOQOL-100 Danish validation study provided QoL assessment in 257 individuals consisting of 4 patient groups with mental and physical disorders. The participating groups comprised individuals with: (1) schizophrenic disorder or depression, (2) diabetes mellitus, (3) severe chronic physical illness, such as arthritis, heart disease and hypertension, (4) gynaecologic disorders and (5) a group of healthy controls. The analysis revealed adequate internal consistency of the instrument (*alpha *range: 0.88 to 0.95 across the WHOQOL-100 domains) and satisfactory discriminant validity between the five population groups across the WHOQOL-100 domains. The differences between these groups were statistically significant (*p *< 0.0001) apart from the domain of spirituality [42].

Finally, the Canadian version of WHOQOL-100, which was tested with a convenience sample of 144 people, demonstrated satisfactory test/retest and consistency reliability (range: 0.71 to 0.89 across domains) and was able to differentiate between healthy and ill populations, providing support for construct validity [40].

## Conclusion

Investigation into the basic psychometric properties of the WHOQOL-100 Greek version produced satisfactory results. It is worth noting that the WHOQOL *environment *domain did not contribute strongly as a component in the QoL measurement. This finding was observed in other validation studies, showing that the ability of the WHOQOL to distinguish between and across populations is mainly observed in the *physical health *and *psychological health *domains rather than in the *environment *and *social relationships *domains [20,51,60].

Although no significant differences were identified at the *environment *domain level between physically ill and mentally ill participants, differences were observed at the facet level. Thus, specific environmental issues seem to bear particular value for the mentally ill, such as the facet of *financial resources *and, for those hospitalised, the facet of *physical safety and security*. It is argued that this finding may reflect the fact that patients' perceptions and evaluations of these specific environmental facets could be critically influenced by their health status, whereas other facets referring to home environment, could be assessed independently of the respondents' health condition. Also, certain facets, as for example the facet of *acquiring new knowledge and skills*, may be age dependent identifying younger individuals. It would be useful to investigate such hypotheses with suitable cohorts.

Finally, it is noted that the domain of *social relationships *has shown the ability to discriminate significantly between psychiatric and non psychiatric clinical populations, both at the domain and facet level. Regarding the *physical health *domain, ability to distinguish between patient groups was not strong at the domain level but it was evident in four out of seven facets. It is suggested that this domain may be of particular interest for future investigation in specifically selected groups with distinct differences in their physical well-being, as well as in the way they perceive their illness and condition of health.

Regarding limitations, it is noted that methodological issues in the present study can be raised, as is the case with several other WHOQOL validation studies. As mentioned earlier, WHO guidelines for new language versions [16,41] were followed throughout the present study. Validation studies may use different methodologies, as for example selecting groups that could be equivalently controlled for social demographic data as in control clinical studies. In the current study, the participating groups were recruited on convenience. Thus, differences were expected to appear in terms of sample sizes, as well as age and gender across groups. Provided that a matched control group methodology was adopted, the QoL differences observed between the participating groups are likely to be more robust. Thus, controlling sociodemographic variables would enhance investigation across specific diagnostic populations, which was however beyond the scope of the present validation study.

Overall, the WHOQOL-100 Greek version has demonstrated good reliability and internal consistency, converging well with similar measurements, whilst successfully differentiating between different patient groups. It is an instrument that may be used to measure treatment benefits reflected on QoL changes in Greek patients, providing healthcare professionals with important and even crucial patient-reported measurements. WHOQOL-100 assessment may contribute to the growing crosscultural study of QoL, particularly in groups of patients for whom a more extended QoL investigation is needed, as in the case of developing patient-focused services and health policies. Additionally, such outcomes are useful not only for individual patient monitoring, but also for service evaluation of mental and physical healthcare, as well as crosscultural comparisons. It is noted that as clinical trials expand internationally, it is essential to develop instruments that measure quality of life across cultures in a valid and reliable way.

## Competing interests

The authors declare that they have no competing interests.

## Authors' contributions

MGC: conception, design, data collection, analysis and interpretation, preparation of manuscript. ET: data collection, analysis and interpretation, preparation of manuscript. VT: interpretation and editing. IAL: analysis, interpretation and comments on first draft. GNC: design, interpretation, comments on first draft, editing. GNP: comments on the final draft, editing.
